# Exploring risk factors for re-hospitalization in a psychiatric inpatient setting: a retrospective naturalistic study

**DOI:** 10.1186/s12888-022-04472-3

**Published:** 2022-12-22

**Authors:** Isabella Berardelli, Salvatore Sarubbi, Elena Rogante, Denise Erbuto, Mariarosaria Cifrodelli, Carlotta Giuliani, Giuseppa Calabrò, David Lester, Marco Innamorati, Maurizio Pompili

**Affiliations:** 1 Department of Neurosciences, Faculty of Medicine and Psychology, Suicide Prevention Centre, Mental Health and Sensory Organs, Sant’Andrea Hospital, Sapienza University of Rome, Via Di Grottarossa, 1035, 00189 Rome, Italy; 2 Department of Human Neurosciences, Sapienza University of Rome, Viale Dell’Università, 30, 00185 Rome, Italy; 3 Psychiatry Residency Training Program, Faculty of Medicine and Psychology, Sapienza University of Rome, Sant’Andrea Hospital, Psychiatry Unit, Via Di Grottarossa, 1035, 00189 Rome, Italy; 4 Psychology Program, Stockton University, Galloway, NJ USA; 5 Department of Human Sciences, European University of Rome, Via Degli Aldobrandeschi 190, 00163 Rome, Italy

**Keywords:** Suicide risk, Re-hospitalization, Hospitalization, Prevention strategies

## Abstract

**Background:**

The reduction of multiple psychiatric hospitalizations is an important clinical challenge in mental health care. In fact, psychiatric re-hospitalization negatively affects the quality of life and the life expectancy of patients with psychiatric disorders. For these reasons, identifying predictors of re-hospitalization is important for better managing psychiatric patients. The first purpose of the present study was to examine the readmission rate in a large sample of inpatients with a psychiatric disorder. Second, we investigated the role of several demographical and clinical features impacting re-hospitalization.

**Method:**

This retrospective study enrolled 1001 adult inpatients (510 men and 491 women) consecutively admitted to the University Psychiatric Clinic, Sant'Andrea Hospital, Sapienza University of Rome between January 2018 and January 2022. To identify risk factors for psychiatric re-hospitalization, we divided the sample into 3 subgroups: the Zero-Re group which had no readmission after the index hospitalization, the One-Re group with patients re-admitted only once, and the Two-Re with at least two re-admissions.

**Results:**

The groups differed according to previous hospitalizations, a history of suicide attempts, age at onset, and length of stay. Furthermore, the results of the regression model demonstrated that the Two-Re group was more likely to have a history of suicide attempts and previous hospitalizations.

**Discussion:**

These results indicate the importance of assessing risk factors in psychiatric hospitalized patients and implementing ad hoc prevention strategies for reducing subsequent re-hospitalizations.

## Introduction

The reduction of multiple psychiatric hospitalizations is an important clinical challenge in mental health care [1, 2]. Research shows that the rates of psychiatric re-admissions vary from 10% to more than 80% [3–5], and re-hospitalization can vary from one month later [5–7] to seven years later [8]. Psychiatric re-hospitalization negatively affects the quality of life and the life expectancy of patients with psychiatric disorders [9]. Psychiatric re-hospitalization is often associated with severe psychological distress for both patients and their families, a worse course for the illness, and a loss of social and employment functioning [1, 10].

The high readmission rates to psychiatric hospitals have given birth to the concept of “revolving door users” or ‘‘high-frequency users’’ to describe those patients who are frequently admitted to hospital and remain functioning after release only for short periods of time [11]. Previous studies have demonstrated that almost 1 in 7 psychiatric patients are re-hospitalized within 30 days of discharge and re-hospitalization rates vary in different countries [12–18]. The CEPHOS-LINK study [19] reported that re-hospitalization rates in Italy were the lowest in all of Europe, and a recent study on this topic [20] reported that the incidence of re-admissions of psychiatric patients in Italy was 16%. Therefore, identifying predictors of re-hospitalization is important for improved management of psychiatric patients. Donisi, et al. reviewed 58 studies [21] and found that the most important predictors of psychiatric re-admission were previous hospitalizations and being unemployed. In another review of 26 studies, Zanardo et al. [22] found that being young, single, with less social support and a previous involuntary admission were the most important factors predicting psychiatric re-hospitalization. Conversely, Sfetcu, et al. [23] found that medication adherence and compliance with follow-up appointments were protective factors. It is well known that several patient characteristics are associated with psychiatry re-hospitalization, including psychiatric diagnosis [24–26], concomitant use and abuse of different substances [24], gender [27, 28], age [29], marital status [24, 25, 27], treatment status [25, 26, 29] and higher severity of symptoms at discharge [24]. Furthermore, a history of admissions has been identified as a strong predictor of re-admission [24–28, 30–32].

Suicide risk (suicide ideation and attempts) is one of the primary reasons for psychiatric hospitalization [33, 34], and a history of suicide attempts is one of the most powerful predictors of later attempts [35]. Data on the relation between single and multiple suicide attempters and risk of re-hospitalization are still controversial [36–38]. Recently, Cepeda et al. [39] observed that the risk of re-hospitalization for suicide risk (suicide ideation and attempts) improves during the first month after the initial hospitalization, and about half of hospitalizations for suicide risk occurred in the first 3 months after the initial hospitalization. Furthermore, the authors showed that re-hospitalization for suicidal ideation or suicide attempt within a year ranges from 7.96% to 11.24%.

The first purpose of the present study was to examine the re-admission rate in a large sample of patients in a psychiatric inpatient setting. The second purpose was to investigate the role of several demographical and clinical features impacting re-hospitalization. Based on the research discussed above, we hypothesize that re-hospitalized psychiatry inpatients may have a higher risk of suicide attempts and a higher risk of future psychiatric hospitalization and that they constitute a population that is clinically difficult to manage. Specifically, we assessed: (1) to what extent suicide attempt and suicidal ideation may predict re-hospitalization; (2) the possible role of psychiatric diagnosis, age at onset of psychiatric illness, the presence of previous hospitalizations in psychiatric settings, the presence of substance use, the length of stay and the type of admission (voluntary or compulsory) for psychiatric re-hospitalization.

## Materials and methods

### Participants

The participants included in this retrospective study were 1001 adult inpatients (out of 1600 patients) hospitalized in the Psychiatric Diagnosis and Care Service (PSDC) of the University Psychiatric Clinic, Sant'Andrea Hospital, Sapienza University of Rome, between January 2018 and January 2022).

From the 1001 patients who participated in the study, there were 510 men and 491 women. The mean age of the participants was 40.50 years (standard deviation = 15.1; age-range = 18–90 years). The sociodemographic and clinical characteristics of the sample at index admission are summarized in Table 1. Inclusion criteria were: (1) any psychiatric disorder according to the Diagnostic and Statistical Manual of Mental Disorders (DSM-5) [40] requiring hospitalization (psychiatric inpatients), and (2) informed consent for participation in the study provided by the patient. Exclusion criteria were: (1) severe neurological disorders (epilepsy, cognitive impairment, or genetic syndromes) (2) the presence of cognitive deficits causing linguistic and comprehension problems, and (3) incomplete clinical records. We excluded 599 patients who presented with neurological and cognitive disorders or missing data.

**Table 1:** Characteristics of the sample at admission index

Variable	Whole Sample	Re-admission			Statistical test	*p*-value
	**(*N* = 1001)**	**Zero**	**One**	**Two or more**		
		**(*N* = 790)**	**(*N* = 132)**	**(*N* = 79)**		
*At the admission index*						
Age M ± SD	42.50 ± 15.1	42.77 ± 15.0	43.57 ± 15.7	38.01 ± 15.2	F_2,998_ = 3.95	.020
Sex (female), n(%)	491 (49.1%)	398 (50.4%)	50 (37.9%)	43 (54.4%)	χ^2^_2_ = 8.07	.018
Previous hospitalizations, n(%)	534 (55.9%)	382 (51.0%)	91 (71.1%)	61 (77.2%)	χ^2^_2_ = 33.83	** < .001**
History of suicide attempts, n(%)	167 (17.4%)	111 (14.8%)	28 (21.7%)	28 (35.4%)	χ^2^_2_ = 23.21	** < .001**
Suicidal ideation, n(%)	304 (30.4%)	231 (29.2%)	45 (34.1%)	28 (35.4%)	χ^2^_2_ = 2.30	.316
Suicide attempt, n(%)	146 (14.6%)	124 (15.7%)	13 (9.8%)	9 (11.4)	χ^2^_2_ = 3.81	.149
Psychiatric diagnosis					χ^2^_8_ = 9.38	.312
Schizophrenia or other psychoses, n(%)	322 (32.2%)	255 (32.3%)	38 (28.8%)	29 (36.7%)		
Bipolar disorders, n(%)	213 (21.3%)	162 (20.5%)	37 (28.0%)	14 (17.7%)		
Depressive disorders, n(%)	138 (13.8%)	117 (14.8%)	14 (10.6%)	7 (8.9%)		
Personality disorders, n(%)	139 (13.9%)	104 (13.2%)	21 (15.9%)	14 (17.7%)		
Others, n(%)	189 (18.9%)	152 (19.2)	22 (16.7%)	15 (19.0%)		
Substance use, n(%)	195 (19.5%)	142 (18.0%)	27 (20.5%)	26 (32.9%)	χ^2^_2_ = 10.31	.006
Type of admission, n(%)(compulsory)	199 (19.9%)	159 (20.1%)	20 (15.2%)	20 (25.3%)	χ^2^_2_ = 3.35	.187
Post-discharge destination					χ^2^_2_ = 0.39	.824
Home, n(%)	216 (21.6%)	173 (22.0%)	28 (21.2%)	15 (19.0%)		
Clinical settings, n(%)	783 (78.4%)	615 (78%)	104 (78.8%)	64 (81.0%)		
Age at psychiatric disorder onset M ± SD	28.28 ± 13.7	28.98 ± 13.5	27.66 ± 14.5	22.79 ± 12.3	F_2,887_ = 7.17	** < .001**
Length of stay (days) M ± SD	10.26 ± 9.6	9.65 ± 9.4	11.29 ± 7.7	14.67 ± 13.3	H_2_ = 27.21	** < .001**

Based on the number of re-admission after the index, we divided the sample into 3 subgroups: the Zero-Re-admission group (Zero-Re) which had no readmission after the index hospitalization, the One-Re-admission group (One-Re) with patients re-admitted only once, and the Two-Re-admission (Two-Re) with more than one re-admission.

The Italian model of psychiatric care is organized to provide personalized treatment, chosen according to the prognosis for and characteristics of the psychiatric disorder, but also in relation to the patient’s psychological and social resources. The principal aim of this is to reduce the need for hospitalization to a minimum and to limit its duration. In Italy, the Psychiatric Diagnosis and Care Service (PSDC) provides for the needs of patients requiring medical treatment involving a stay in hospital, in the case of both voluntary admissions and compulsory treatment. It also guarantees emergency treatment in conjunction with the hospital's emergency department. After discharges from the psychiatric ward, patients, with a good prognosis and improving characteristics of the psychiatric disorders, along with social and relational resources, can be admitted to intermediate residential or semi-residential units or to mental health outpatient services.

The assessment of psychiatric patients with particular attention to suicide risk is part of several investigations approved by the local ethics review board of Sant’Andrea Hospital –Sapienza University of Rome. In addition, the study analyzed the demographical and clinical characteristics of patients as part of a broader investigation approved by the local Institutional Review Board of Sant’Andrea Hospital – Sapienza University of Rome, on admission. The study was conducted according to the guidelines of the Declaration of Helsinki. All participants provided informed consent for participation in the study. Additionally, all patients were in full possession of their faculties and capable of understanding instructions and willing to participate, while patients with cognitive deficits were not included in the study.

### Measures

Three independent psychiatrists (MC, CG and GC) at the University Psychiatric Clinic, Sant'Andrea Hospital, Sapienza University of Rome, analyzed each clinical record. Data were recorded using a structured checklist created for this study by the authors. In the checklist, researchers included psychiatric diagnoses, current and past suicidal ideation and current and past suicide attempt, age at onset of psychiatric illness, the presence of previous hospitalizations in psychiatric settings, the presence of substance use, the length of stay, the type of admission (voluntary or compulsory), and the type of care after discharge. Researchers collected these data through the clinical history provided by the patient, the family members and other psychiatrists who followed the patients during the course of the illness. Moreover, researchers investigated data on both current and past hospitalizations.

GC, IB, MC and CG made the psychiatric diagnosis during the first days of hospitalization based on the Diagnostic and Statistical Manual of Mental Disorders, fifth edition [40] and supported by the Structured Clinical Interview for DSM-5 Disorders (SCID-5) [41]. The authors mentioned above assessed suicidal ideation and suicide behavior according to the definition adopted by Posner et al. [42, 43] in the Columbia–Suicide Severity Rating Scale (C-SSRS). Suicidal ideation included thoughts about a wish to be dead or active thoughts of wanting to end one’s life [42, 43]. Furthermore, suicide attempt was defined as a nonfatal self-directed, potentially injurious behavior with an intent to die that may or may not have resulted in injury [44, 45]. The assessment based on the definition provided by C-SSRS was conducted for all the patients admitted to the Psychiatric Diagnosis and Care Service (PSDC) of the University Psychiatric Clinic, Sant'Andrea Hospital, Sapienza University of Rome.

An individual's first psychiatric admission was considered the index admission in the study period. We included only admissions lasting longer than 48 h because clinical information collected for shorter admissions is limited. We considered psychiatric re-admission to be any unplanned admission to an acute psychiatric unit for a psychiatric reason [20]. Further re-admission in addition to the index admission was considered only during the study period (from January 2018 to January 2022). Data regarding re-hospitalization included the type of admission (compulsory or voluntary), and the presence of suicide ideation and attempt.

### Statistical analysis

All statistical analyses were performed with the Statistical Package for Social Sciences (SPSS 27.0) [46]. A series of ANOVAs, chi-square (χ^2^), and One-Way Fisher exact tests were used for bivariate analyses. One-way Fisher exact tests and chi-squared (χ^2^) tests were used for the 2 × 2 and N x N contingency tables, respectively. The Bonferroni correction was applied to correct for multiple testing. In case of non-normality, the Kruskal–Wallis test was used. Tamhane's T2 *post-hoc* tests were used for group comparisons at the index admission. Significant variables in the bivariate analyses were then included as independent variables in a multinomial regression analysis model with groups as a criterion.

## Results

### Group characteristics

Of the whole sample of 1001 psychiatric inpatients, 790 patients (78.9%) were not re-hospitalized during the period of the study (Zero-Re), and 211 patients (21.1%) were re-hospitalized (Re-H). Specifically, 132 patients (13.2%) were re-hospitalized once (One-Re), and 79 patients (7.9%) were re-hospitalized at least twice (Two-Re). At admission index, twenty-one percent of the patients suffered from bipolar disorders, 13.8% depressive disorders, 32.2% schizophrenia or other psychoses, 13.9%, personality disorders, and 18.9% other specified disorders (mostly anxiety disorders). One hundred ninety-five patients also reported substance use (19.5%). Previous hospitalizations were present for 534 patients (53.3%), and 167 patients (16.7%) had a history of suicide attempts. About twenty percent of patients had a compulsory admission. Most of the patients (78.9%) were discharged to clinical settings (i.e., community health services). At admission index, suicidal ideation and a suicide attempt were observed in 303 patients (30.3%) and 133 patients (13.3%), respectively (Table 1). Among re-hospitalized patients (*n* = 211), 69 patients (32.7%) had suicidal ideation and 33 patients (15.7%) had attempted suicide at the time of re-hospitalization (Table 2).

**Table 2:** Characteristics of the groups One-Re-admission vs Two-Re-admissions

Variable	One-Re (*N* = 132)	Two-Re (*N* = 79)	One-way Fisher Exact Test
Suicidal ideation	43 (32.6%)	26 (32.9%)	1.0^a^
Suicide attempt	20 (15.2%)	13 (16.5%)	.846^a^
Type of admission (compulsory)	24 (18.2%)	17 (21.5%)	.592^a^

### Difference between groups

The Zero-Re, One-Re, and Two-Re groups differed according to previous hospitalization (χ^2^_2_ = 33.83, *p* < 0.001), a history of suicide attempts (χ^2^_2_ = 23.21, *p* < 0.001), age at onset (F_2,887_ = 7.17, *p* < 0.001), and length of stay (H_2_ = 27.21, *p* < 0.001). Moreover, the groups differed according to age (F_2,998_ = 3.95, *p* = 0.020), sex (χ^2^_2_ = 8.07, *p* = 0.018), and substance use (χ^2^_2_ = 10.31, *p* = 0.006). The three groups did not differ in terms of diagnosis, admission type, the type of destination after discharge, and suicide ideation and attempt at the index admission (Table 1). Moreover, no differences were found for suicide ideation and attempt and admission type at the time of re-hospitalization.

Specifically, patients re-hospitalized at least two times (Two-Re) had a lower age at onset (22.79 ± 12.3 vs. 27.66 ± 14.5, and 28.98 ± 13.5, respectively for Zero-Re and One-Re patients’ groups). Moreover, the Two-Re group had a longer length of stay at the index admission (14.67 ± 13.3 vs. 9.65 ± 9.4) and were more likely to have a history of a suicide attempts (35% vs. 14.8%, for Zero-Re) compared to Zero-Re patients. Both One-Re and Two-Re groups were more likely to have a previous hospitalization compared to the Zero-Re group (71.1% and 77.2% vs. 51%, respectively).

A multinomial logistic regression model with groups as a criterion that used significant variables at the bivariate analysis as independent variables explained 10% of the between-group variance (Nagelkerke *R*^2^ = 0.096; − 2LL = 1150.77; χ^2^_8_ = 65.10, *p* < 0.001). Overall, previous hospitalizations (χ^2^_2_ = 23.87, *p* < 0.001), a history of suicide attempts (χ^2^_2_ = 6.74, *p* < 0.05), age at onset (χ^2^_2_ = 7.92, *p* < 0.05), and length of stay (χ^2^_2_ = 8.57, *p* < 0.05) were significantly and independently associated with group differences (Table 3). Compared to the Zero-Re group, the One-Re patients were more likely to have a previous hospitalization (OR = 2.28; 95% CI = 1.48/3.51). The Two-Re patients were more likely to have a previous hospitalization (OR = 3.13; 95% CI = 1.68/5.82), a history of suicide attempts (OR = 2.09; 95% CI = 1.21/3.62), and a longer length of stay (OR = 1.03; 95% CI = 1.01/1.05), but had a lower age at onset (OR = 0.97; 95% CI = 0.95/0.99) compared to No-Re patients.

**Table 3:** Multinomial regression analysis

		**No re-admission**			**No re-admission**		
		**vs**			**vs**		
		**One re-admission**			**Two or more re-admission**		
**Variable**	**χ**^**2**^_**2**_	**b (SE)**	**OR**	**95% CI OR**	**b (SE)**	**OR**	**95% CI OR**
Previous hospitalization	23.87^***^	0.82 (0.22)^***^	2.28	[1.48–3.51]	.99 (0.30)^***^	2.86	[1.48–4.87]
History of suicide attempts	6.74^*^	0.21 (0.26)	1.24	[0.75–2.05]	0.74 (0.28)^**^	2.09	[1.21–3.62]
Age at psychiatric disorder onset	7.92^*^	-0.02 (0.01)	0.99	[0.98–1.01]	-0.31 (0.01)^**^	0.97	[0.95–0.99]
Length of stay (days)	8.57^*^	0.01 (0.01)	1.01	[0.99–1.03]	0.03 (0.01)^**^	1.03	[1.01–1.05]

Note. Nagelkerke *R*^2^ = 0.096,-2LL = 1102.05. Model χ^2^_8_ = 65.10, *p* < .001. *p < .05, **p < .01, ***p < .001

## Discussion

The present study was designed to determine the readmission rate and to assess risk factors for psychiatric re-hospitalization in psychiatric inpatient settings.

Considering the total sample of 1001 psychiatric inpatients, 211 were re-hospitalized (Re-H, 21.1%) and 790 were not (Zero-Re, 78.9%). Patients re-hospitalized at least two times were younger than other groups and had a lower age at onset of the psychiatric disorder. Moreover, the Two-Re group had a longer length of hospitalization and had a history of suicide attempts more frequently as compared to the Zero-Re patients. Both the One-Re and Two-Re groups were more likely to have previous hospitalization compared to the Zero-Re patients. Furthermore, the three groups did not differ in current suicide ideation, suicide attempt and psychiatric diagnosis at the index admission. The demographical and clinical characteristics of the Two-Re group (younger and with a lower age at onset of the psychiatric disorder) are in line with previous studies on this topic suggesting a higher severity of psychiatric symptoms with possible clinical relapse and, therefore, more complex clinical management [47–49]. Furthermore, these results suggest that illness severity, more than the type of psychiatric diagnosis, could be related to specific hospitalization characteristics (length of hospitalization and re-hospitalization) probably in relation to the clinician’s perceived need for hospitalization [50]. Moreover, regarding the presence in the Two-Re group of a history of suicide attempts, probably clinicians tend to hospitalize patients with a history of suicide attempts more frequently, due to the peculiar characteristics of this population of patients, the difficulty of clinical management and the fear of possibly more lethal future suicide attempts [51, 52].

In the present study the overall incidence of re-hospitalization was 21.1%. This result appears to be different from those observed in previous studies [16–19]. The reasons for the different rate of re-hospitalizations in the present study could be due to the well-defined area of our study, regional and local specificities for our psychiatric ward (such as disparities in the availability of psychiatric beds and differences in post-discharge community-based care services) and the period of observation.

Among the various clinical factors involved in re-hospitalization evaluated in the study, the results of the regression model demonstrated that the Two-Re group were more likely to have a previous suicide attempt and hospitalization than did the Zero-Re group. It is well known, in fact, that about 40% of suicide attempters die as a result of their second or later attempt and 80% die within a year of the first attempt [35]. Furthermore, people who have attempted suicide multiple times show different demographical and clinical differences compared to one-time attempters, which is related to a higher lethality of the subsequent suicidal behavior [51, 53]. All these features make patients with multiple suicide attempts a group of patients at higher risk of re-hospitalization. These results indicate the importance of assessing multiple domains of impairment for estimating the risk for future suicidal behavior and future hospitalizations. Since, among patients recently discharged from psychiatric hospitalization, suicide death and suicide attempt rates are far higher than in the general population [54], re-hospitalization because of suicidal ideation or suicidal attempts could be used as an objective outcome to measure the effectiveness of treatments for patients at risk for suicide or of strategies for suicide prevention [55].

Finally, the results of the present study partially confirm previous research on re-hospitalized patients regarding age and age at symptom onset and longer length of stay [56, 57]. Furthermore, both the One-Re and Two-Re groups were more likely to have previous hospitalizations compared to Zero-Re group, suggesting that re-hospitalized patients present a more complex psychiatric symptomatology. As suggested by Jaramillo-Gonzalez et al. [58], in re-hospitalized patients the association between duration of hospitalization and re-hospitalization may be due to the difficulty in managing patients with severe psychiatric disorders who usually present comorbidity and less response to pharmacological treatments, and that may explain the longer hospital stays and the need of future rehospitalizations.

The present study has several limitations. This was a retrospective naturalistic study and, therefore, the assessment was limited to retrospective data. The differences in subgroups size may have affected the statistical power of the analyses. We did not use psychometric assessment tools to evaluate the severity of psychiatric symptoms or to assess other psychiatric dimensions that could be involved in re-hospitalization. Psychiatric variables at re-hospitalization were limited and we considered only patients re-hospitalized in our psychiatric ward. Moreover, as predictors of re-admission can be different at different time intervals following discharge, it would have been appropriate to establish different time points for the evaluation of re-admissions. Finally, this research did not include the re-admission period, which could indicate the risk to the so-called psychiatric revolving door, and people in the sample might have been hospitalized in other psychiatric wards, and so the readmission rate reported in the present study cannot be generalized.

In conclusion, the results of the present study suggest that identifying patients at risk of re-hospitalization could help predict future re-hospitalization and facilitate the design of ad hoc prevention strategies, including screening to identify at-risk individuals, psychoeducation on the management of mental disorders, easy access to psychiatric emergence units, treatment interventions, and follow-up care after psychiatric hospitalization.

## Data Availability

The datasets used and/or analyzed during the current study are available from the corresponding author on reasonable request.
